# Antibiotic Resistance in India: Drivers and Opportunities for Action

**DOI:** 10.1371/journal.pmed.1001974

**Published:** 2016-03-02

**Authors:** Ramanan Laxminarayan, Ranjit Roy Chaudhury

**Affiliations:** 1 Center for Disease Dynamics, Economics & Policy, Washington, D.C., United States of America; 2 Princeton Environmental Institute, Princeton University, Princeton, New Jersey, United States of America; 3 Public Health Foundation of India, New Delhi, India; 4 Apollo Hospitals Educational and Research Foundation, New Delhi, India

## Abstract

Ramanan Laxminarayan and Ranjit Roy Chaudhury examine the factors encouraging the emergence of antibiotic resistance in India, the implications nationally and internationally, and what might be done to help.

**Summary Points**

- Antibiotic use is a major driver of resistance. In 2010, India was the world’s largest consumer of antibiotics for human health.
- Access to antibiotics is rising, which portends well for the large proportion of India’s population that has thus far had poor access to these life-saving drugs.
- The convergence of factors such as poor public health infrastructure, rising incomes, a high burden of disease, and cheap, unregulated sales of antibiotics has created ideal conditions for a rapid rise in resistant infections in India.
- Over-the-counter, nonprescription sales of carbapenems in India are among the highest in the world and contribute to growing carbapenem resistance among Gram-negative organisms.
- Improving regulation of drug production and sales, better managing physician compensation, and encouraging behavior change among doctors and patients are of immediate priority.

## Antibiotic Resistance and Use in India

Antibiotic resistance is a global public health threat [1], but nowhere is it as stark as in India [2]. The crude infectious disease mortality rate in India today is 416.75 per 100,000 persons (author calculations based on World Bank data and the Global Burden of Disease, 1990 [1,2]) and is twice the rate prevailing in the United States when antibiotics were introduced (roughly 200 per 100,000 persons) [3]. A mix of poor public health systems and hospital infection, high rates of infectious disease, inexpensive antibiotics, and rising incomes is coming together to increase prevalence of resistant pathogens and is increasing the burden of untreatable neonatal sepsis and health-care-associated infections [4].

New Delhi metallo-β-lactamase (NDM) enzymes, first reported in 2008, are now found worldwide[5]. In India, *Escherichia coli* (*n* = 1,815) isolated from the community showed high overall resistance to ampicillin, naladixic acid, and co-trimoxazole (75%, 73%, and 59%, respectively) between 2004 and 2007 [6]. Nearly a third of isolates are resistant to injectables like aminoglycosides (represented by gentamicin). From 2008 to 2013, *E*. *coli* resistance to third-generation cephalosporins increased from 70% to 83%, and fluoroquinolone resistance increased from 78% to 85%. Ten percent of *E*. *coli* isolates were resistant to carbapenems in 2008, increasing to 13% in 2013 [7]. Among *Klebsiella pneumoniae* isolates, third-generation cephalosporin resistance decreased from 90% to 80%, and fluoroquinolone resistance increased from 57% to 73% [7]. Carbapenem resistance among *K*. *pneumoniae* increased from 2% in 2002 to 52% in 2009 in one tertiary-care hospital in New Delhi [8].

Resistance to fluoroquinolones among invasive *Salmonella* Typhi isolates in India increased from 8% in 2008 to 28% in 2014. However, resistance in 2014 to two older antibiotics—ampicillin, 5%, and cotrimoxazole, 4%—is decreasing, possibly because of a decline in consumption of these two drugs, and is much lower than rates of resistance to fluoroquinolones. Resistance to nalidixic acid in *S*. Typhi is increasing (resistance is about 20%–30%) because of widespread use of other quinolones and not because of nalidixic acid use per se. Among *Enterococcus faecium* isolates, 11% were vancomycin resistant [9].

Surgical site infections are a problem and are predominantly related to Gram-negative pathogens. A recent study from Mumbai reported a 1.6% rate of surgical site infections, with 66% caused by Gram-negative bacilli (GNB) [10]. With diminishing options for treating multidrug-resistant *Acinetobacter baumannii* and other resistant infections, colistin use is increasing, but resistance to colistin is on the rise [10]. Gram-positive infections are also a problem. High rates of methicillin-resistant *Staphylococcus aureus* (MRSA) in clinical isolates from various studies in India have been documented, with rates as high as 54.8% (ranging between 32% and 80%) recorded [11]. A recent report records a steep increase in MRSA, from 29% of *S*. *aureus* isolates in 2009 to 47% in 2014 based on data from a large private laboratory network [7].

The overall burden of resistance is hard to assess for the general population but is likely focused on neonates and the elderly, both of whom are more prone to infections and vulnerable to ineffective treatment. Although accurate estimates of the overall burden of resistance are not available, it is estimated that 58,000 neonatal deaths are attributable to sepsis caused by drug-resistance to first-line antibiotics each year [4].

### Antibiotic Use

Antibiotic use is a major driver of resistance. In 2010, India was the world’s largest consumer of antibiotics for human health at 12.9 x 10^9^ units (10.7 units per person). The next largest consumers were China at 10.0 x10^9^ units (7.5 units per person) and the US at 6.8 x10^9^ units (22.0 units per person) [11]. Seventy-six percent of the overall increase in global antibiotic consumption between 2000 and 2010 was attributable to BRICS countries, i.e., Brazil, Russia, India, China, and South Africa [11]. In BRICS countries, 23% of the increase in the retail antibiotic sales volume was attributable to India, and up to 57% of the increase in the hospital sector was attributable to China.

**Fig 1:**
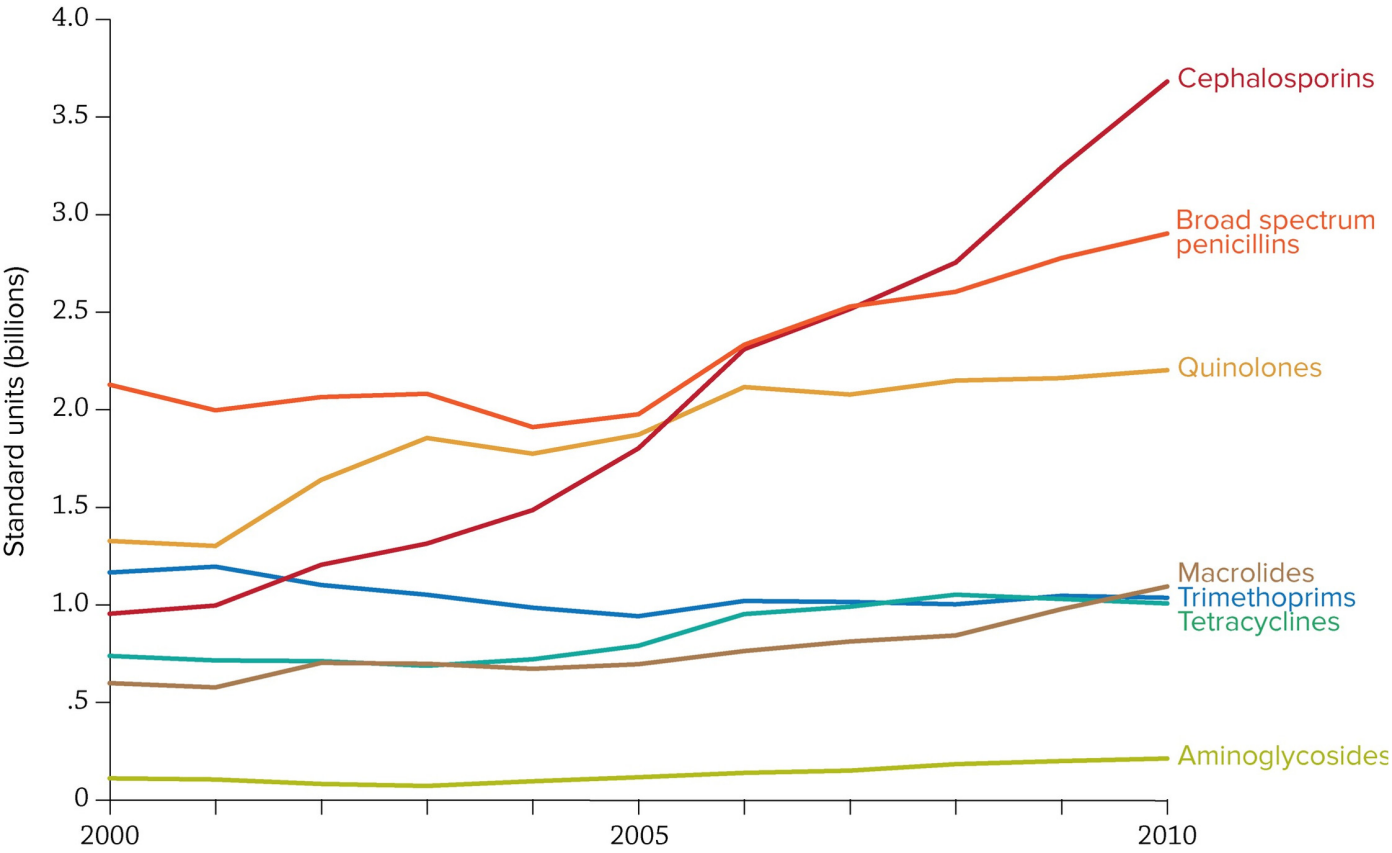
Trends in antibiotic consumption in India, 2000–2010. The data used to create this figure can be accessed at the Center for Disease Dynamics, Economics & Policy (CDDEP) ResistanceMap website at http://resistancemap.cddep.org/resmap/c/in/India.

Overall, ampicillin and co-trimoxazole use is declining in India (Fig 1), while quinolone consumption is high and increasing in India. Rates of carbapenem use per capita are low compared to other antibiotics in 2000 but had risen to over 10 million standard units by 2010 [11].

The scale-up in antibiotic use in India has been enabled by rapid economic growth and rising incomes, which have not translated into improvements in water, sanitation, and public health, although evidence exploring this key issue is anecdotal [12]. Antibiotics continue to be prescribed or sold for diarrheal diseases and upper respiratory infections for which they have limited value [10,13]. India’s large population is often blamed for the easy spread of resistant pathogens, but population densities in India are lower than those in parts of Indonesia or China. The main problem is that India lags on basic public health measures. Immunization rates (as measured by diphtheria-tetanus-pertussis [DPT3]) coverage in India (72%) lag behind those in Brazil (95%), China (99%), and Indonesia (85%). The percentage of the population with access to improved sanitation facilities in India (36%) was far lower than the percentage in Brazil (81.3%), China (65.3%), and Indonesia (58.8%) [14]. Under the Swacch Bharat Abhiyan (Clean India Program), the government has committed to providing toilets and improving sewage systems, but these measures will take time to implement.

### Health System Factors

Health system factors are also at fault. Doctors routinely receive compensation from pharmaceutical companies and pharmacists in exchange for antibiotic prescriptions [15]. Infection control in hospitals is poorly monitored and could be improved. A point prevalence study in a large tertiary care hospital in India found an overall health-care-associated infection prevalence of 7%, with a third of these being surgical site infections [16]. Half of all patients were receiving antimicrobials.

Over-the-counter access to antibiotics is a problem, but regulations to restrict access have to be balanced against the need to maintain access for the significant proportion of the population that lacks access to doctors. Indeed, lack of access to effective and affordable antibiotics still kills more children in India than does drug resistance [4]. However, to prevent over-the-counter (OTC) sales of important antibiotics, the Central Drugs Standard Control Organization (CDSCO) implemented Schedule H1 in India starting March 1, 2014. The H1 list includes 24 antibiotics, such as third- and fourth-generation cephalosporins, carbapenems, antituberculosis drugs, and newer fluoroquinolones. Antibiotics have previously been listed under Schedule H, which contained drugs that could be sold only with a valid prescription; almost all antibiotics were easily available over the counter in the country, leading to their rampant use. The stricter Schedule H1 specifies that the drugs covered by it carry a prominent Rx symbol in red and contain a box with red borders with a printed warning on their packaging. Moreover, drugs included in Schedule H1 can only be sold with the prescription of a registered medical practitioner and require that that pharmacist maintain a separate register with the patient’s name, contact details of the prescribing doctor, and the name and dispensed quantity of the drug. The register has to be retained for at least three years and is subject to audit by the government. There have been some instances of enforcement of Schedule H1, with licenses of 213 retail pharmacies in the division canceled for “non-compliance to dispensing medicines without prescription and giving bill” in some parts of India [17]. Several important antibiotics, including gentamicin, piperacillin-tazobactam, linezolid, and tigecycline, are not included in Schedule H1. There is potential for OTC sales of these antibiotics by pharmacists to compensate for the restricted sales of stronger prescription antibiotics.

The problem of resistance is exacerbated by a wide range of fixed-dose combinations in the market, often without scientific or medical merit or evaluation. A recent study reported 48 fixed dose combinations and 22 loose antimicrobials for tuberculosis [18]. Loose antimicrobials come without packaging and do not mention the name of the drug, its manufacturer, the date of manufacture, or the date of expiry. There is poor clinician awareness of the rationality and dosing of fixed-dose combinations [19]. Incorporating principles of antimicrobial stewardship and appropriate use into undergraduate and postgraduate medical education can be implemented and is under consideration by the Government of India. A more difficult problem is that of regulating the sales of substandard and illegitimate antimicrobials, the extent of which is poorly quantified [20].

### Antibiotics in the Environment

Environmental antibiotic pollution encourages the transfer of resistance genes to human commensal and pathogenic bacteria [21]. In particular, waste water treatment plants serving antibiotic manufacturing facilities have been implicated in the transfer of resistance genes into human microbiota and pose a serious threat to antibiotic effectiveness given the size of India’s pharmaceutical sector [22]. There are no regulations governing the discharge of antimicrobial waste into the environment, and these are needed.

Growing antibiotic use in the animal sector is resulting in a greater selection of pathogens [23] and is being driven by increased demand for meat and poultry. The extreme growth in consumption of chickens is primarily the result of the expansion of this sector in India alone, where areas of high consumption (30 kg per km^2^) are expected to grow 312% by 2030 [23]. A recent Organisation for Economic Co-operation and Development (OECD) report indicated that the costs of withdrawing antimicrobial growth promoters in India would be roughly US$1.1 billion [24]. However, widespread resistance may hold more consequence for India than for other countries because of India’s high bacterial disease burden. Currently, India does not have regulatory provisions for the use of antimicrobials in cattle, chickens, and pigs raised for domestic consumption. Recent studies in various regions of India have discovered antimicrobial residues in food animal products (such as chicken meat and milk) [25], indicating that antibiotic use in food animal production is widespread. There are no standards for tolerance of antibiotic residues in poultry, although such standards do exist for seafood—including shrimps, prawns, or any other variety of fish and fishery products—under the Food Safety and Standards (Contaminants, Toxins, and Residues) Regulations of 2011; more recently, standards for honey have also been developed [26]. Effective limits on antimicrobial growth promoters in India could have knock-on effects on neighbors such as Bangladesh, Nepal, and Sri Lanka that are likely to be guided and influenced by regulatory action in India, given the interconnectedness of the region’s pharmaceutical commerce [27].

## Conclusion

Poor public health indicators, rising incomes, and the availability of inexpensive antibiotics over the counter without a prescription are converging to create the ideal conditions for a large-scale selection and dissemination of resistance genes in India. India is not alone in this battle, and the experiences of other countries in dealing with antimicrobial resistance are described in the most recent State of the World’s Antibiotics Report (2015) [9].

On the positive side, efforts by groups like the Indian Association of Pediatrics, the Global Antibiotic Resistance Partnership [2], and the Chennai Declaration [28] have helped build awareness about the problem among professional bodies, the media, policy makers, and the lay public. A high-level committee has been convened to address the issue at the Ministry of Health and is expected to issue recommendations soon.

A few urgent priorities for immediate implementation stand out. First, improved capacity of drug regulatory bodies is essential to safeguard against powerful antibiotics being sold over the counter and to phase out the use of antimicrobial growth promoters in livestock. These capabilities are also needed to ensure the safety and reliability of India’s pharmaceutical manufacturing sector, which now supplies a significant proportion of the world’s pharmaceutical needs. Second, behavior change is needed among physicians and patients. India has achieved remarkable reductions in smoking in buildings and workplaces through regulation and behavior change communication [29]. Similar campaigns could work to educate the public and physicians about the dangers of uncontrolled antibiotic use, as has been the case in high-income countries, but more research is needed to see how well this could work in India [30]. Third, changes in rules under which physicians can accept compensation are already in place under the rules of the Medical Council of India, and should be extended to cover prescriptions for antibiotic sales. Fourth, there are little data on the extent of resistance, with the exception of a few single-hospital reports, despite the need for robust national data in order to drive policy. A national surveillance platform is being built under ResistanceMap (www.resistancemap.org), a global data repository for antimicrobial use and resistance, which relies on reports from accredited laboratory service providers [9]. The Indian Council of Medical Research has established a National Programme on Antimicrobial Surveillance in ten laboratories based at academic centers and covering priority pathogens identified by the World Health Organization. When complete, this network will focus on (i) diarrhea (e.g., *Shigella*, *Vibrio cholerae*), (ii) enteric fever (e.g., *S*. Typhi, *S*. Paratyphi), (iii) sepsis caused by Enterobacteriaceae (e.g., *E*. *coli*, *K*. *pneumoniae*), (iv) other Gram-negative organisms (e.g., *Pseudomonas aeruginosa*, *A*. *baumannii*), (v) Gram-positive bacteria (e.g., MRSA and vancomycin-resistant enterococci [VRE]), (vi) fungal infections (e.g., *Candida* spp.), and (vii) respiratory pathogens (e.g., *Streptococcus pneumoniae*). This can be supplemented through period point prevalence surveys that can provide period data at a fairly low cost. Alongside this, better surveillance data on antibiotic consumption are needed.

Fifth and finally, India should phase out antimicrobial growth promoters from livestock when these drugs are medically important and when these are premixed with feed. Such a move would have regional consequences and would send a strong signal of the country’s commitment to tackle this issue.

## Abbreviations

BRICS: Brazil, Russia, India, China, and South Africa
CDDEP: Center for Disease Dynamics, Economics & Policy
CDSCO: Central Drugs Standard Control Organization
DPT3: diphtheria tetanus pertussis
GNB: Gram-negative bacilli
MRSA: methicillin-resistant *Staphylococcus aureus*
NDM: New Delhi metallo-β-lactamase
OECD: Organisation for Economic Co-operation and Development
OTC: over the counter
VRE: vancomycin-resistant enterococci
